# 

**Published:** 1890-12-06

**Authors:** 


Ditto per Annum, 8s., post free.

### THE HOSPITAL

A WEEKLY JOURNAL OF

### Science, Medicine, Nursing and Philanthropy.

### SATURDAY, DECEMBER 6th, 1890.

**Crude Science** . . . . 149
**Passing Topics.**—The National Pension Fund and Workers.—III. . . . . 150
**The Doctor's Armchair.**—Has a New Day Dawned? . . 151
**Working Women in Large Towns.** XVII.—Aids for Working Women . . . . 152
**The Workhouse Infirmary Nursing Association.**—Interview with Miss J. Wilson . . . . 153
**The Lords and the Hospitals** . . . . 154
**Work and Food.** By Sir Andrew Clark, Bart., M.D., F.R.S. 154
**Everybody's Page** . . . . 155
**Editor's Letter Box**—Northerner and Southerner . . 155
**Notes and News** . . . . 156
**December** . . . . 162
**Annotations.**—The Sunday Fund—The Steady Briton—Paupers and their Food . . . . 158
**The Practitioner's Mirror and Retrospect:**—
The Pathological Society of London.—On Cancer—On the Non-Contagiousness of Cancer . . . . 159
The London Post-Graduate Course at the Paddington Infirmary—Pathology . . . . 160
New Drugs, Appliances, and Things Medical.—Corsets or Not? 161; Spectacle Appliances . . . . 162
**Scraps and Gleanings** . . . . 163
**Books Received** . . . . 163
**The Student of Medicine.**—Editorial Notes—Appointments—Students' Medical Societies—Notes from the Schools—Pass Lists—Athletics . . . . 164

### EXTRA SUPPLEMENT—THE NURSING MIRROR.

**En Passant.**—Short Items—Nursing Heroines—Rare Courage of a Nurse—Belfast Nurses' Home—Nurses' Food xlvii

**Lectures on Surgical Ward Work and Nursing.** By Alex. Miles, M.B. (Edin.), C.M., F.R.C.S.E.—Lecture VI.—Dressings . . . . xlviii

**Nursing Medals and Certificates.** No. I.—The Royal Red Cross . . . . xlix

**For Reading to the Sick.**—The Duty of Cheerfulness xlix

**Nurses on Their Travels.**—American Notes . . . l

**The Trained Nurse in the Nursery** . . . l

**Everybody's Opinion.**—The General Bonus Fund of the National Pension Fund for Nurses— . . . li

**Presentations—Notes and Queries** . . . li

**Domestic Medicine in the Eighteenth Century** . lii

**Where to Go—Appointment** . . . lii

**Amusements and Relaxation—Lectures** . . . lii

